# Identification of saturated and unsaturated 1-methoxyalkanes from the Thai millipede *Orthomorpha communis* as potential “Raincoat Compounds”

**DOI:** 10.1038/s41598-018-30156-8

**Published:** 2018-08-06

**Authors:** Aem Nuylert, Yasumasa Kuwahara, Tipparat Hongpattarakere, Yasuhisa Asano

**Affiliations:** 1 Asano Active Enzyme Molecular Project, JST, ERATO, 5180 Kurokawa, Imizu, Toyama, 939-0398 Japan; 2 Biotechnology Research Center and Department of Biotechnology, Toyama Prefectural University, 5180 Kurokawa, Imizu, Toyama, 939-0398 Japan; 3 Department of Industrial Biotechnology, Faculty of Agro-Industry, Prince of Songkla University, Hat Yai, Songkhla, 90112 Thailand

## Abstract

Mixtures of saturated and unsaturated 1-methoxyalkanes (alkyl methyl ethers, representing more than 45.4% of the millipede hexane extracts) were newly identified from the Thai polydesmid millipede, *Orthomorpha communis*, in addition to well-known polydesmid defense allomones (benzaldehyde, benzoyl cyanide, benzoic acid, mandelonitrile, and mandelonitrile benzoate) and phenolics (phenol, *o*- and *p*-cresol, 2-methoxyphenol, 2-methoxy-5-methylphenol and 3-methoxy-4-methylphenol). The major compound was 1-methoxy-*n*-hexadecane (32.9%), and the mixture might function as “raincoat compounds” for the species to keep off water penetration and also to prevent desiccation.

## Introduction

Certain arthropods are well known to produce exocrine secretions which serve a variety of functions such as defense against predators^1^, antimicrobial and antifungal activities^2^, protection against moisture^3^, and intraspecific information pheromones^4–6^. Millipedes (Diplopoda) belonging to seven of the 16 orders (composed of 145 families, over 12,000 species described) possess exocrine glands (repugnatory glands or ozadenes, located on the pleurotergites) and the chemical compositions of their secretions have been studied for more than 140 species^7–10^. Among them, 58 species of Polydesmida have been examined worldwide and their defense allomone compositions have been well documented^7–9^. Most polydesmid species are cyanogenic, and their defense components are mainly produced by two enzymes [hydroxynitrile lyase (HNL)^11^ and mandelonitrile oxidase (MOX)^12^] from a mandelonitrile substrate stored in the reservoir of repugnatory glands. HNL is responsible for the production of HCN and benzaldehyde, which is further reduced to benzyl alcohol or oxidized to benzoic acid. MOX is responsible for the oxidation of mandelonitrile into benzoyl cyanide and the production of hydrogen peroxide as a by-product^13^. Benzoyl cyanide reacts chemically with mandelonitrile to give mandelonitrile benzoate^14^, and also with water to produce benzoic acid and HCN. Polydesmida also contains non-cyanogenic species, *Eutrichodesmus elegans* and *E. armatus*, whose defensive allomones have been identified as (*Z*)- and (*E*)-2-nitroethenylbenzene and 2-nitroethylbenzene^15,16^. Those nitro compounds are also known to be derived from L-phenylalanine, the same precursor of mandelonitrile^15^.

Contrary to Insecta where they have been well documented^3^, no waxy compounds, such as hydrocarbons and fatty acid esters, have ever been described as agents for possible protection against moisture and desiccation among most of the Diplopoda^5,6^. They occur on all continents (except Antarctica) and in nearly all terrestrial environments from the temperate to the tropical zones^17^, and only some species of millipedes (mentioned later) belonging to Julida have been known to possess certain fatty acid esters, presumably as cuticular lipids.

**Figure 1:**
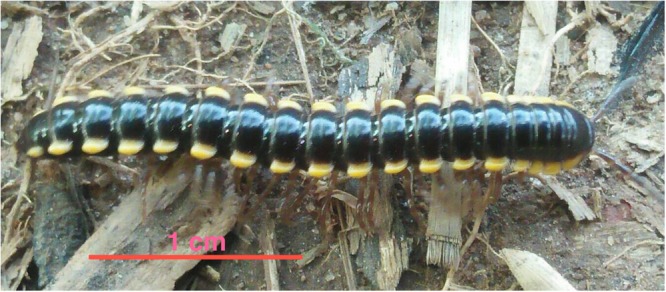
Picture of the Thai millipede *O. communis*.

The Thai millipede *Orthomorpha communis* Likhitrakarn, Golovatch & Panha, 2011 (Paradoxosomatidae: Polydesmida) is distributed widely in the eastern part of Thailand close to the border with Cambodia and has newly been found in Hat Yai District, Songkhla Province, Thailand. The live coloration of blackish-brown body rings, with paraterga and epiproct showing a distinctive creamy yellow during contracting (Fig. 1).

Using gas chromatography coupled with mass spectrometry (GC/MS) analysis of the species, we happened to detect a series of saturated and unsaturated wax-like components, other than the conventional mixtures derived from mandelonitrile. These wax-like components have not been reported in other millipedes, and our hypothesis is that they have a function similar to the “raincoat” found in the oribatid mite, *Liacarus subterraneus* (Acari: Oribatida). This mite secretes di-glycerides via an esterification reaction between fatty acids and glycerin^5^. As a result, the chemical structures of these saturated and unsaturated wax-like components was investigated.

## Results

### GC/MS analyses of the Thai millipede

**Figure 2:**
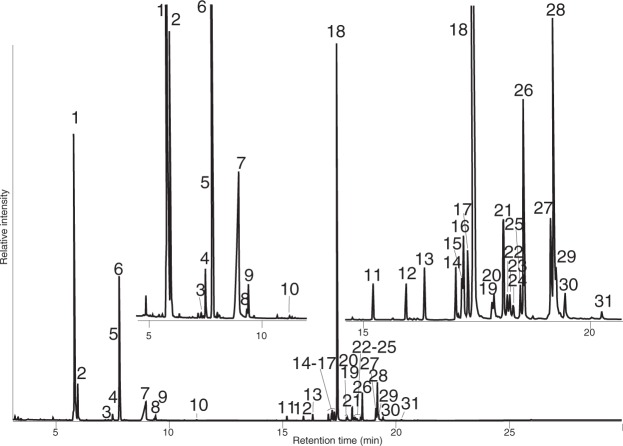
GC profile of the hexane extract from the Thai millipede *O. communis*. 1–10 and 26; components known as defensive allomone of polydesmid millipedes, 11–25 and 27–31; waxy compounds. Structure and names see Fig. 4 and Table 1.

As shown in Fig. 2, the GC-profile showed a total of 31 peaks (=compounds) including non-resolved components [2-methoxyphenol (**5**) and benzoyl cyanide (**6)**, as mentioned later].

Mass spectra of all peaks are summarized in Table 1, including identified results by co-chromatography with authentic compounds and (or) elucidated structures, as mentioned later.

**Table 1:** Gas chromatographic and mass spectral data of compounds from extracts of the Thai millipede, *O. communis*.

Peak no.	Retention		Content	Mass spectrometric fragmentation (m/z)	Compound identified	
	time	index*	(%)		as	Standard obtained (Library ID %)
1	5.838	959	26.8	106 (M^+^, 100), 105 (96), 77 (90), 51 (32)	benzaldehyde	com. (97%)
2	5.972	970	3.1	94 (M^+^, 100), 66 (30.4)	phenol	com. (91%)
3	7.185	1068	0.1	108 (M^+^, 100), 107 (87), 90 (21), 89 (13), 80 (11), 79 (28), 77 (27)	*o*-cresol	com.
4	7.512	1094	0.6	108 (M^+^, 81), 107 (100), 90 (7), 79 (20), 77 (22)	*p*-cresol	com. (96%)
5	7.819	1118	0.2	124 (M^+^, 94), 109 (100), 81 (44), 65 (5), 53 (15)	**2-methoxyphenol	com. (96%)
6	7.822	1119	14.9	131 (M^+^, 72), 105 (100), 77 (55), 74 (8), 51 (23)	benzoyl cyanide	com. (93%)
7	8.982	1213	6.0	122 (M^+^, 85), 105 (100), 77 (72), 51 (35)	benzoic acid	com. (96%)
8	9.340	1242	0.1	138 (M^+^, 77), 123 (100), 95 (25), 77 (12), 67 (21), 55 (11), 51 (9)	2-methoxy-5-methylphenol	com. (94%)
9	9.405	1247	0.5	138 (M^+^, 96), 123 (100), 95 (26), 77 (8), 67 (13), 55 (10), 51 (8)	3-methoxy-4-methylphenol	94%
10	11.225	1394	trace	133 (M^+^, 70), 115 (39), 105 (100), 77 (95), 51 (45)	mandelonitrile	com. (90%)
11	15.203	1716	0.3	196 (M^+^−32, 26), 168 (15), 140 (8), 125 (20), 111 (39), 97 (72), 83 (92), 69 (71), 55 (60), 45 (100)	1-methoxy-*n*-tetradecane	syn. (90%)
12	15.930	1775	0.3	210 (M^+^−32, 20), 195 (4), 182 (18), 167 (3), 154 (19), 139 (6), 125 (15), 111 (40), 97 (70), 83 (92), 69 (90), 56 (96), 45 (100)	1-methoxy-13-methyl-tetradecane	
13	16.341	1808	0.5	210 (M^+^−32, 25), 182 (13), 154 (6), 140 (8), 125 (21), 111 (46), 97 (81), 83 (100), 69 (73), 55 (61), 45 (98)	1-methoxy-*n*-pentadecane	
14	17.033	1864	0.5	224 (M^+^−32, 11), 209 (2), 196 (9), 181 (3), 168 (8), 153 (5), 140 (12), 125 (18), 111 (49), 97 (72), 83 (100), 69 (95), 55 (94), 45 (97)	1-methoxy-14-methylpentadecane	
15	17.159	1874	0.5	254 (M^+^, 2), 222 (10), 194 (2), 180 (1), 166 (2), 152 (2), 137 (6), 123 (12), 109 (31), 96 (62), 82 (100), 67 (73), 55 (47), 45 (39)	1-methoxy-*n*-hexadecene	
16	17.203	1878	1.0	254 (M^+^, 2), 222 (14), 194 (3), 179 (1), 166 (3), 152 (3), 137 (8), 123 (16), 109 (30), 96 (71), 82 (100), 67 (59), 55 (61), 45 (45)	1-methoxy-*Z*−9-hexadecene	syn
17	17.300	1885	0.7	254 (M^+^, 2), 222 (11), 194 (2), 180 (1), 166 (2), 152 (3), 137 (7), 123 (14), 109 (27), 96 (74), 82 (100), 67 (54), 55 (78), 45 (49)	1-methoxy-*E*−9-hexadecene	
18	17.466	1899	32.9	224 (M^+^−32, 31), 213 (1), 196 (15), 182 (2), 168 (5), 154 (7), 139 (10), 125 (27), 111 (53), 97 (88), 83 (100), 69 (69), 55 (59), 45 (86)	1-methoxy-*n*-hexadecane	syn. (91%)
19	17.832	1928	0.2	268 (M^+^, 2), 236 (16), 208 (3), 194 (1), 180 (2), 166 (1), 152 (3), 137 (9), 123 (19), 109 (34), 96 (66), 82 (100), 67 (54), 55 (55), 45 (42)	1-methoxy-*Z*-9-15-methyl-hexadecene	
20	17.872	1932	0.4	238 (M^+^−32, 2), 223 (1), 210 (2), 196 (1), 182 (1), 169 (9), 153 (22), 140 (11), 125 (44), 111 (66), 97 (94), 83 (93), 71 (89), 69 (65), 57 (100), 45 (68)	1-methoxy-methyl-hexadecane	
21	18.082	1948	1.1	238 (M^+^−32, 13), 223 (2), 210 (11), 195 (3), 182 (10), 168 (2), 154 (6), 139 (5), 125 (14), 111 (37), 97 (57), 83 (100), 69 (74), 57 (78), 45 (80)	1-methoxy-15- methylhexadecane	
22	18.166	1955	0.3	238 (M^+^−32, 5), 227 (1), 209 (15), 181 (7), 168 (4), 153 (6), 139 (12), 125 (28), 111 (44), 97 (80), 83 (83), 70 (100), 57 (62), 55 (56), 45 (66)	1-methoxy-14-methylhexadecane	
23	18.220	1960	0.3	268 (M^+^, 2), 236 (13), 208 (2), 194 (1), 180 (2), 166 (2), 152 (3), 137 (9), 123 (16), 109 (31), 96 (72), 82 (100), 67 (56), 55 (56), 45 (37)	1-methoxy-*Z*-9-heptadecene	syn.
24	18.295	1966	0.2	268 (M^+^, 2), 236 (7), 208 (3), 193 (2), 180 (3), 166 (3), 151 (5), 137 (9), 123 (17), 109 (32), 96 (72), 82 (100), 67 (59), 55 (68), 45 (48)	1-methoxy-*E*-9-heptadecene	
25	18.454	1979	0.4	238 (M^+^−32, 20), 227 (1), 210 (10), 196 (1), 182 (4), 168 (4), 154 (5), 139 (8), 125 (24), 111 (48), 97 (85), 83 (100), 69 (68), 57 (56), 45 (82)	1-methoxy-*n*-heptadecane	
26	18.530	1985	2.3	237 (M^+^, 17), 116 (39), 105 (100), 89 (11), 77 (25), 51 (11)	mandelonitrile benzoate	prepd. (80%)
27	19.126	2033	1.2	280 (M^+^, 2), 248 (3), 219 (1), 205 (1), 191 (1), 184 (3), 177 (2), 163 (4), 152 (9), 149 (9), 135 (25), 121 (30), 109 (30), 95 (69), 81 (89), 67 (100), 55 (51), 45 (47)	1-methoxy-*Z,Z*-9,12-octadecadiene	syn.
28	19.195	2038	3.2	282 (M^+^, 2), 250 (12), 222 (2), 208 (1), 194 (2), 180 (1), 166 (2), 152 (3), 137 (9), 123 (16), 109 (29), 96 (70), 82 (100), 67 (54), 55 (54), 45 (37)	1-methoxy-*Z*-9-octadecene	syn. (99%)
29	19.241	2042	0.8	282 (M^+^, 3), 250 (14), 239 (1), 222 (2), 208 (1), 194 (2), 180 (2), 165 (2), 152 (4), 137 (10), 123 (18), 109 (31), 96 (80), 82 (100), 69 (49), 55 (58), 45 (38)	1-methoxy-*E*-9-octadecene	
30	19.438	2058	0.4	252 (M^+^−32, 19), 241 (1), 224 (8), 210 (1), 196 (3), 182 (3), 168 (4), 154 (5), 139 (9), 125 (26), 111 (49), 97 (86), 83 (100), 69 (68), 57 (58), 45 (78)	1-methoxy-*n*-octadecane	syn.
31	20.251	2124	0.1	296 (M^+^, 1), 264 (5), 236 (2), 222 (1), 208 (2), 194 (1), 180 (2), 165 (2), 151 (4), 137 (9), 123 (16), 109 (28), 96 (71), 82 (100), 69 (55), 55 (70), 45 (45)	1-methoxy-*Z*-9-nonadecene	

*Retention index; caluculated by HP-5 column under conditions described in text, as reported (Kovat. 1958),

**Detectable by selected ion chromatography com.; commercially avilable, syn.; prepared from palm oil, prepd; by synthesis.

A total of 20 peaks (11, 12, 13, 14, 15, 16, 17, 18, 19, 20, 21, 22, 23, 24, 25, 27, 28, 29, 30, and 31, 45.4%) were newly detected in the present species and represented the first discovery of the hereafter named “waxy compounds” among Polydesmida. Their structures were later elucidated as 1-methoxyalkanes by (1) column chromatography (SiO_2_) behavior, (2) NMR analysis, and (3) GC-mass spectra (the presence of M^+^ or M^+^-32, and m/z 45), using 1-methoxyalkanes prepared from palm oil as an authentic compound.

### Chromatographic behavior of waxy compounds on SiO_2_ column

**Figure 3:**
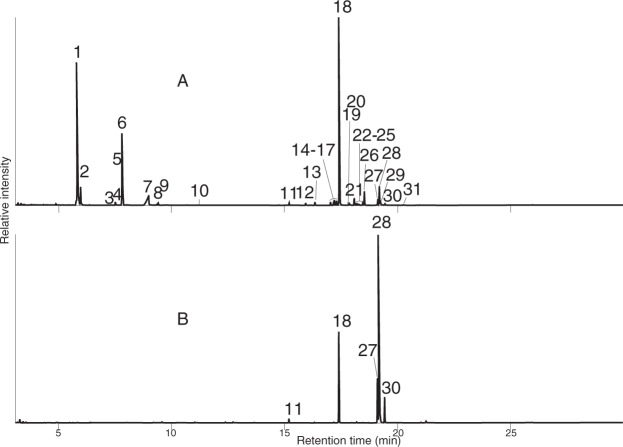
Comparison of GC profiles. (**A**) from Thai millipedes, and (**B**) 1-methoxyalkanes prepared from palm oil. Structure and names, see Fig. 4 and Table 1.

When the hexane extracts from seven adult millipedes were separated by silica gel column (300 mg, 0.5*ϕ* × 2.1 cm) chromatography, most of the waxy compounds were recovered in the fraction (3 ml) eluted with the 1% Et_2_O in hexane, indicating their non-polar nature (less polar than esters). Methyl ether mixtures prepared from palm oil were also recovered in the same 1% Et_2_O in hexane fractions, as mentioned above. Peak 1 was eluted by 2% Et_2_O in hexane (3 ml) with the remaining waxy compounds. As shown in Fig. 3, peaks 11, 18, 27, 28, and 30 derived from the palm oil were identical to those derived from millipedes using GC t_R_s and mass spectra (mentioned below).

### ^1^H-NMR spectrum of the waxy compounds from millipedes and 1-methoxyalkanes from palm oil

The NMR spectrum of millipede compounds after being purified by the SiO_2_ column proved to be almost the same as the spectra for the 1-methoxyalkanes found in palm oil, except for a difference in the integral on long chain methylenes. The following chemical shifts were observed for millipedes; ω-methyl (3H, *t*, J = 6.6 Hz at δ 0.88 ppm), long chain methylenes (26H, *m* at δ 1.52–1.60 ppm), -CH_2_-CH_2_-O- (2H, *m*, at δ 1.52–1.60 ppm), CH_3_-O- (3H, s, at δ 3.33 ppm), and -CH_2_-CH_2_-O (2H, *t*, J = 6.6 Hz at δ 3.36 ppm), thus, identifying the structure as 1-methoxy-hexadecane. As a result, waxy components from millipede were shown to be a mixture of saturated and unsaturated 1-methoxyalkanes.

### GC/MS analysis of standard 1-methoxyalkanes prepared from palm oil

**Figure 4:**
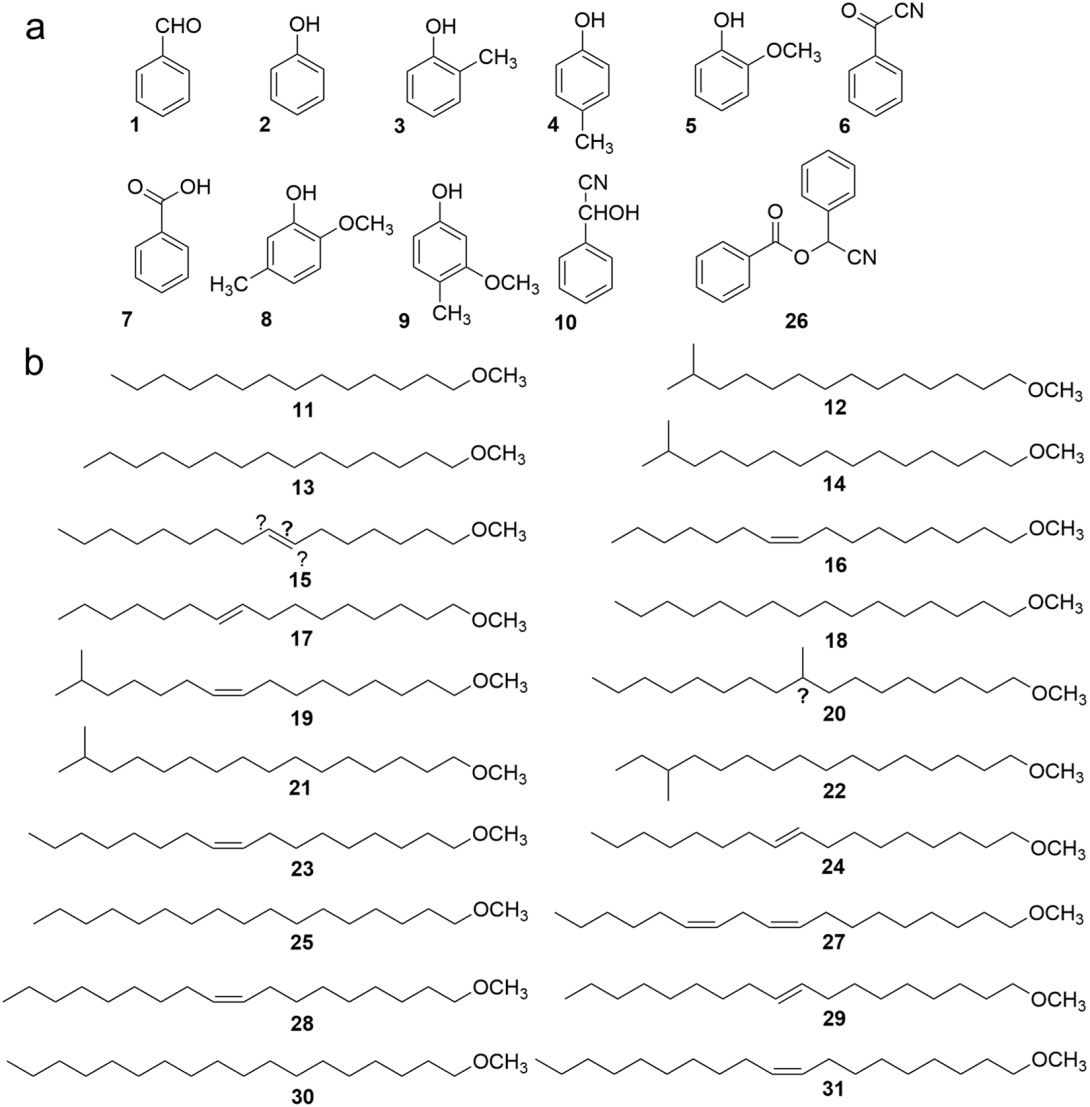
Chemical structures identified or elucidated in the hexane extract of the Thai millipede *O. communis*. (**a**) Compounds commonly detectable among polydesmid millipedes as defense allomone, (**b**) 1-methoxyalkanes, newly detected in the species.

Peaks 11, 18, 27, 28, and 30 in the preparation from the palm oil showed the same GC-retention times and mass spectra to those from millipedes (this corresponded to 84.4% of the waxy composition in the millipede). Among them, significant results (more than 90% identities) of library search results were available for peaks 11 (90%), 18 (91%) and 28 (99%). As a result, structures of peaks 11, 18, and 28 were identified as 1-methoxy-*n*-tetradecane (**11**), 1-methoxy-*n*-hexadecane (**18**) and 1-methoxy-*Z*-9-octadecene (**28**), respectively (Fig. 4).

Based on the above results, the other two peaks 27 and 30 were thought to be similar to the 1-methoxyalkanes originated from the fatty acid compositions of the palm oil, and described as 1-methoxy-*Z,Z*-9,12-octadecadiene (**27**) and 1-methoxy-*n*-octadecane (**30**) by mass spectra analysis (Table 1, mentioned later).

### Structure elucidation of waxy compounds by GC/MS

Mass spectra of waxy compounds mostly indicated alkene series of fragment ions with sigmoid intensity from m/z 41 to m/z 139 or m/z 153. The fragment ion at m/z 45 (-CH_2_^+^-OMe) was always observable in all compounds, indicating the presence of oxygen in the molecule. Saturated compounds (**11**, **12**, **13**, **14**, **18**, **20**, **21**, **22**, **25**, and **30**, except **20** and **22**) displayed each reliable intensity (more than 11%) of fragment at m/z M^+^-32(MeOH) with trace intensity fragment at M^+^-1, but no M^+^ ion, and m/z 83 was constantly observable as the base ion peak, respectively. On the other hand, in the case of monoene compounds (**15**, **16**, **17**, **19**, **23**, **24**, **28**, **29**, and **31**), M^+^ ions were detected with M^+^-32(MeOH) and the base ion at m/z 82. In the case of dienoic compound (**27**), the base ion was observed at m/z 67. As a whole, these facts implied that elimination of CH_3_-OH or H^+^ could occur in all those components, and each compound was elucidated as 1-methoxyalkane (**11**, **12**, **13**, **14**, **18**, **20**, **21**, **22**, **25**, and **30**), 1-methoxyalkene (**15**, **16**, **17**, **19**, **23**, **24**, **28**, **29**, and **31)**, or 1-methoxyalkadiene (**27**) (Table 1).

### Structure elucidation of 1-methoxyalkanes in the millipede

Mass spectra of **13** and **25** were identical to those of **11** [M^+^-32(MeOH) ions at m/z 196], **18** [M^+^-32(MeOH) ions at m/z 224] and **30** [M^+^-32(MeOH) ions at m/z 252] derived from palm oil reduction, except M^+^-32(MeOH) ions observed at m/z 210 and 238. Therefore, peak 13 was described as 1-methoxy-*n*-pentadecane (**13**) and peak 25 as 1-methoxy-*n*-heptadecane (**25**) (Table 1, Fig. 4).

Peaks 12, 14, 20, 21, and 22 indicated M^+^-32 (MeOH) ions at m/z 210, m/z 224, m/z 238, m/z 238, and m/z 238 as the largest fragments, respectively, and these were thought to be 1-methoxy-methyl-branched (substituted) alkanes. Differences on Kovat’s retention indexes (∆KI between 1-methoxy-*n*-alkane and 1-methoxy-methyl substituted alkane = increment of additional methyl residue) were calculated as follows; KI of peak 12 (1775) was that of peak 11 (1716) + ∆KI 59, likewise ∆KI 56 for peak 14, ∆KI 33 for peak 20, ∆KI 49 for peak 21 and ∆KI 56 for peak 22. If we assume peak 12 and peak 14 are 1-methoxy-13-methyltetradecane (**12**, *iso*-C_15_ ether, ∆KI 59) and 1-methoxy-14-methylpentadecane (**14**, *iso*-C_16_ ether, ∆KI 56), respectively, peak 21 (∆KI 49) or 22 (∆KI 56) should be 1-methoxy-15-methylhexadecane (**21**, *iso*-C_17_ ether), while peak 20 (∆KI 33) remained obscure as a branched C_17_-OMe. If ∆KI′ is compared between *n-* and *iso*-alkyl series, ∆KI′ between peaks 12 and 13 was observed as 33, likewise between peaks 14 and 18 as 35, and between peaks 21 and 25 as 31, then 21 was more likely a 1-methoxy-15-methylhexadecane (**21**, iso-C_17_ ether), because ∆KI′ between peaks 22 and 25 was 24. On the other hand, mass fragment M^+^-32 (MeOH)-15 (CH_3_) ion was observed in peaks 12 (4%), 14 (2%), 20 (1%), 21(2%) and 22 (1%) suggested the presence of methyl residue in the molecule, while peak 22 gave strong M^+^-32 (MeOH)-29 (CH_3_CH_2_) ion (18%), indicative of a removable ethyl residue (*ante-iso* carbon chains) in the structure. As a whole, peaks 12, 14, and 21 should be proposed as 1-methoxy-*iso*-alkanes; 1-methoxy-13-methyltetradecane (**12**, *iso*-pentadecyl methyl ether), 1-methoxy-14-methylpentadecane (**14**, *iso*-hexadecyl methyl ether) and 1-methoxy-15-methylhexadecane (**21**, *iso*-heptadecyl methyl ether), respectively. Peak 22 was subsequently characterized as 1-methoxy-14-methylhexadecane (**22**, *ante-iso*-heptadecyl methyl ether). Peak 20 was supposed to be 1-methoxy-methyl-branched hexadecane (**20**, methyl-branched hexadecyl methyl ether), whose methyl position remained obscure. Stereo chemistry of each optically active carbon in **20** and **22** remained obscure.

### Structure elucidation of 1-methoxy-alkenes (unsaturated methyl ethers) in the millipede

Based on calculated C=C double bond increment (∆KI = −20) for *Z*−9-ene (peak 28) to saturated (peak 30) of standards derived from palm oil, and likewise (∆KI = −25) for *Z*,*Z*-9,12-diene (peak 27), peak 16 (∆KI = −21) was characterized as 1-methoxy-*Z*-9-hexadecene (**16**, *Z*-9-hexadecenyl methyl ether), and peak 23 (∆KI = -19) was characterized as 1-methoxy-*Z*-9-heptadecene (**23**, *Z*-9-heptadecenyl methyl ether). Calculated KI for putative C_19_-OMe was 2143, then peak 31 (KI = 2124) corresponded to the difference of ∆KI = −19 and the structure of peak 31 was elucidated as 1-methoxy-Z-9-nonadecene (**31**, *Z*-9-nonadecenyl methyl ether). KI value of peak 19 (1928) indicated −20 to that of peak 21 (1946), then the resulting ∆KI = −20 suggested the structure of peak 19 as 1-methoxy-15-methyl-*Z*-9-hexadecene (**19**, *Z*-9-iso-heptadecenyl methyl ether). Although peak 15 looked like 1-methoxy-hexadecene (**15**) by GC/MS, its ∆KI = −25 were larger than Z-9-mono-ene (∆KI = −19 ~ −20), and its C=C bond position remained obscure. Peaks 17, 24, and 29 had almost the same mass spectra as peaks 16, 23, and 28 with ∆KI = −14, −13, and −16 corresponding saturated compounds. Those were proposed as being *E*-isomers, and tentatively elucidated as 1-methoxy-*E*-9-hexadecene (**17**, *E*-9-hexadecenyl methyl ether), 1-methoxy-*E*-9-heptadecene (**24**, *E*-9-heptadecenyl methyl ether), and 1-methoxy-*E*-9-octadecene (**29**, *E*-9-octadecenyl methyl ether). All structures of compounds from *O. communis*, identified, elucidated, or characterized, are presented in Fig. 4.

### Structure elucidation of conventional millipede components

Another five peaks (1, 6, 7, 10, and 26, a total of 50.0%) were conventional polydesmid compounds and identified each as benzaldehyde (**1**), benzoyl cyanide (**6**), benzoic acid (**7**), mandelonitrile (**10**) and mandelonitrile benzoate (**26**), using authentic compounds. The other five peaks (2, 3, 4, 5, and 8, in total 4.1%**)** were also found among Polydesmida and identified as phenol (**2**), *o*-cresol (**3**), *p*-cresol (**4**), 2-methoxyphenol (**5**) and 2-methoxy-5-methylphenol (**8**), using authentic compounds. Peak 9 (0.5%) was elucidated as 3-methoxy-4-methylphenol (**9)** by comparison with MS database (Table 1).

## Discussion

The present polydesmid millipede has recently revealed the possession of a large amount of waxy compounds (mentioned later) in addition to the conventional chemical defense systems represented by five polydesmid compounds{**1**, **6**, **7**, **10**, and **26**, produced by two enzymes; hydroxynitrile lyase (HNL)^11^ and mandelonitrile oxidase (MOX)^12^ from **10** as the common substrate with subsequent chemical and biochemical reactions} and of six phenolic compounds (**2**, **3**, **5**, **8**, and **9**). Then the species corresponds to one of HCN and H_2_O_2_ emitter^13^.

The structures of waxy compounds were elucidated and identified as saturated, unsaturated or methyl branched 1-methoxyalkanes (more than 45.4% of total hexane extracts) by NMR and GC/MS spectra. Most of components (86.6% of total ethers) were identified by co-chromatography with authentic compounds prepared from the palm oil, as follows; **11** (0.7%), **16** (2.2%), **18** (72.5%), **23** (0.7%), **27** (2.6%), **28** (7.0%), and **30** (0.9%). Structures of the other ethers (11.6% of total methyl ethers; **12**, **13**, **14**, **17**, **19**, **21**, **22**, **24**, **25**, **29**, and **31**) were elucidated by calculation using Kovat’s retention index as summarized in Table 1. The position of a double bond in **15** (1.1%) and that of methyl residue in **20** (0.9%) remained obscure. Alkyl moieties of methyl ethers are mostly identical to those of long chain fatty acids, and presumably have those fatty acids as a precursor. At present, there are no other polydesmid millipedes known to possess those waxy compounds, such as hydrocarbons and fatty acid esters, nor other related presumably functioning as “raincoat compounds”.

As far as our data searches are concerned, no other millipedes including Polydesmida or other animals have been reported to produce 1-methoxyalkanes (methyl alkyl ethers)^8,9^, except for the spider *Nephila clavipes* [Arachnida: Araneae]^18^. The major components of the spider silk extracted by pentane or methylene chloride consist of a complex mixture of methyl-branched 1-methoxyalkanes [total 51 compounds, all of those contained up to four methyl groups in each carbon chain (chain length between C_28_ and C_34_)], together with small amounts of hydrocarbons and alcohols^18^.

As a group of waxy components, fatty acid esters have been distributed in several species of millipedes belonging to Julida, other than methyl- and methoxy-substituted benzoquinones^8,9^. A mixture of hexadecyl acetate, Δ9-hexadecenyl acetate and Δ9-octadecenyl acetate has been identified in *Blaniulus guttulatus* [Julida: Blaniulidae]^19^, likewise hexyl oleate and octyl oleate have been identified in *Cibiulus phlepsii* and *Nopoiulus kochii* [Julida: Blaniulidae]^20^. Hexyl esters of alkanoic acids have also been found in *Enantiulus nanus* and *Julus scandinavius* [Julida: Julidae]^21^. A total of 15 esters, composed of fatty acids (C_9_–C_14_, including *iso*- and *ante-iso*-) and *n*-alcohols (C_4_–C_8_) have been identified in *Anaulaciulus* sp. [Julida: Julidae]^22^. However, distribution of hydrocarbons, as waxy components or body surface compounds, has not been reported in millipedes. Two types of unwettability (raincoat effect) due to chemical compounds have been summarized in Liacaridae (Acari, Oribatida), other than physical and morphological “Lotus effect”; (1) a mixture of C_8_–C_10:2_ acid, and their 1,2- and 1-3-di-glycerides, and (2) esters composed of C_15_ and C_16_ acids with C_14_–C_17_ alcohols^23^.

In conclusion, 1-methoxyalkanes (a total of 20 compounds of methyl alkyl ethers, a class of compounds) were discovered as components of hexane extracts in the present species. It is only the second example of its presence in the animal kingdom, and it is presumably present on the body surface as a raincoat to keep off water penetration and also to prevent desiccation. The present discovery of unusual 1-methoxyalkanes in this polydesmid millipede might represent evidence of a chemical evolution of the species that adapts and thrives in heavy tropical rainfalls. Further examples of similar species should be expected.

## Materials and Methods

### Millipedes

The adult species *Orthomorpha communis* Likhitrakarn, Golovatch & Panha, 2011 [Paradoxosomatidae: Polydesmida], often found individually embedded in soils after heavy rains, were manually collected in Banrai (6°54′38.3″N, 100°28′08.0″E), Hat Yai District, Songkhla Province (Southern Thailand), Thailand in December, 2015, and subsequently reared in the laboratory on leaf litters collected from the site under a natural photoperiod with high humidity (ca. 100% RH) at 20 °C. Sizes of adult were as follows; body length 15.0–27.5 mm, a width of midbody pro- and meta-zona 1.1–2.3 and 1.5–3.1 mm, respectively.

### Hexane extraction of millipedes

Five adult millipedes as a group and also one adult millipede (two times) were dipped into *n*-hexane (1 ml, separately) for three minutes, using appropriate glass vials (5 ml volume). Then, the resultant hexane extracts after separation from residual millipede bodies by decantation were each subjected to gas chromatography coupled with mass spectrometry (GC/MS) analyses.

### Analytical methods

GC/MS spectra were obtained as reported^24^ using Hewlett Packard HP 5975 C Inert XL EI/CI MSD with triple-Axis Detector at 75 eV coupled with a 7890 A GC-system equipped with an HP-5 column (30 m × *ϕ* 0.25 mm; 0.25 μm in film thickness) operated in the split-less mode at 60 °C for 2 min, then programmed to increase at 10 °C/min to 290 °C, and finally held at this temperature for 5 min. Helium was used as the carrier gas at a flow rate of 1.00 ml/min. GC and GC/MS data were processed using ChemStation (Hewlett Packard Co.), with reference to an MS database (Wiley 9^th^/NIST 2011 MS Library; Hewlett Packard Co.). In the case of the non-resolved peaks (**5** and **6**), relative amounts were calculated by selected ion chromatography (SIC) using each base ion (m/z 105 and 109, respectively). Retention indices^25^ were calculated under the same GC conditions mentioned above, as described in Bodner and Raspotnig^26^, and were used for structure elucidation. ^1^H-NMR spectra (400 MHz, TMS at δ00.00 as internal standard) were recorded on a Bruker Biospin AC400M spectrometer.

### Chemicals

The following chemicals and solvents were obtained commercially and used as described in the text; two chemicals (**1** and **2**) from Wako Pure Chemical Industries, Japan, two chemicals (**5** and **7**) from Nacalai Tesque Inc., Japan, and five chemicals (**3**, **4**, **6**, **8**, and **10**) from Tokyo Chemical Industry Co., Japan. Compound **26** was prepared as reported previously^16^. Wako Gel C-200, hexane, diethylether (Wako Pure Chemical Industries, Ltd.), lithium aluminum hydride, methyl iodide, and metallic sodium (Junsei Chemical, Japan) were used without purification.

### Preparation of standard 1-methoxyalkane mixtures from palm oil

A mixture of alcohols derived from the palm oil by reduction with lithium aluminum hydride were lead to the corresponding mixture of 1-methoxyalkanes using Williamson’s ether synthesis (reaction between sodium alcoholate and methyl iodide). After addition of water to dissolve solids, the reaction products were extracted by hexane. The hexane extracts, without concentration, was poured on a silica gel column (Wako Gel C-200, 300 mg, 0.5*ϕ* × 2.1 cm in length) prepared without solvent, using a disposable glass pipette (1.5 ml, Iwaki). 1-Methoxyalkanes were obtained as an eluted fraction by 1% ether in hexane.

### Separation of millipede components by a silica gel column

To the silica gel column (Wako C-200, 300 mg) similarly prepared as mentioned above, the hexane extracts (1 ml) from seven millipedes was subjected, and the column was successively eluted with hexane (3 ml), 1% Et_2_O/hexane (3 ml), 2% Et_2_O/hexane (3 ml), 5% Et_2_O/hexane (3 ml), 10% Et_2_O/hexane (3 ml), 50% Et_2_O/hexane (3 ml), and 100% Et_2_O (3 ml). All fractions were subjected to GC/MS analysis.
